# 

**Published:** 1874-07

**Authors:** 


### Table of Contents.

### ORIGINAL COMMUNICATIONS.

Epilepsy of Seventeen Years Standing—Trephined....*By A. W. Calhoun, M.D., Atlanta, Ga*.... 193
Lactation in Advanced Life..*By T. S. Hopkins, M.D., Thomasville, Ga.* 199
Carcinoma of the Stomach, Bowels and Omentum..*By K. P. Moore, M.D., Knoxville, Ga*.... 200
The "Eclectic System" of Obstetrics..*By C. C. Hart, M.D., Cross Keys, Ga*.... 203
Compound Comminuted Fracture of Skull—Case..*By R. Y. Rudicil, M.D., Trion, Ga*.... 204

### MEDICAL SOCIETIES.

Atlanta Academy of Medicine—Malarial Hæmaturia—Curvature of Spine—Vaginal Ovariotomy—Impotency—Absence of Uterus—Physiology of Menstruation—Intestinal Obstructions—Injections Vomited by the Stomach—Ovarian Tumor—Exploratory Incision—United Twins—Dislocated Spleen—Ovarian Adhesions—Chronic Pelvic Abscess—Impermeable Stricture—Abortion—Quickening as a Diagnostic Sign—Acute Glossitis—Mammary Cancer.... 206
American Medical Association at Detroit—Code of Ethics—Professor Sayre on Fractures.... 219

### OUR EXCHANGES.

Erichsen on the Elastic Ligature.... 205
Direct Transfusion from Animal to Man.... 234
Intra-Uterine Medication.... 235
Standard Elixirs, with Numerous formulæ.... 236
Coccyodynia—Syrup of Licorice Root.... 241
Puerperal Convulsions.... 242
Mercury in Syphilis.... 243
Iced-Water Enemata in Dysentery.... 256

### BIBLIOGRAPHICAL.

Prof. Van Buren on the Genito-Urinary Organs and Syphilis.... 245
Hempel on the Science of Homœopathy.... 246
Murray on the Treatment of Cholera.... 249

### EDITORIAL.

Our State Associations.... 250
South-Western Georgia Medical Association.... 254
The Gold-Headed Cane.... 255

